# Ion-Cross-Linked Hybrid Photochromic Hydrogels with Enhanced Mechanical Properties and Shape Memory Behaviour

**DOI:** 10.3390/polym16081031

**Published:** 2024-04-10

**Authors:** Shijun Long, Fan Chen, Han Ren, Yali Hu, Chao Chen, Yiwan Huang, Xuefeng Li

**Affiliations:** 1 Hubei Provincial Key Laboratory of Green Materials for Light Industry, Hubei University of Technology, Wuhan 430068, China; longshijun.hp@163.com (S.L.); chenfan202301@163.com (F.C.); renhann@126.com (H.R.); hu_yali1@163.com (Y.H.);; 2 Hubei Longzhong Laboratory, Xiangyang 441000, China; 3 New Materials and Green Manufacturing Talent Introduction and Innovation Demonstration Base, Hubei University of Technology, Wuhan 430068, China; 4 Hubei Key Laboratory of Polymer Materials, Hubei University, Wuhan 430062, China

**Keywords:** photochromic hydrogels, ion-cross-linked hybrid hydrogels, double network, high mechanical strength, shape memory

## Abstract

Shape-shifting polymers usually require not only reversible stimuli-responsive ability, but also strong mechanical properties. A novel shape-shifting photochromic hydrogel system was designed and fabricated by embedding hydrophobic spiropyran (SP) into double polymeric network (DN) through micellar copolymerisation. Here, sodium alginate (Alg) and poly acrylate-co-methyl acrylate-co-spiropyran (P(SA-*co*-MA-*co*-SPMA)) were employed as the first network and the second network, respectively, to realise high mechanical strength. After being soaked in the CaCl_2_ solution, the carboxyl groups in the system underwent metal complexation with Ca^2+^ to enhance the hydrogel. Moreover, after the hydrogel was exposed to UV-light, the closed isomer of spiropyran in the hydrogel network could be converted into an open zwitterionic isomer merocyanine (MC), which was considered to interact with Ca^2+^ ions. Interestingly, Ca^2+^ and UV-light responsive programmable shape of the copolymer hydrogel could recover to its original form via immersion in pure water. Given its excellent metal ion and UV light stimuli-responsive and mechanical properties, the hydrogel has potential applications in the field of soft actuators.

## 1. Introduction

Hydrogels are soft and wet materials that exhibit a three-dimensional network with good flexibility, hydrophilicity and biocompatibility, which render them ideal as soft actuators. Therefore, stimulus-responsive hydrogels have attracted considerable attention in various fields [1] owing to their promising application prospects [2], including information recording [3], biosensors [4], actuators [5], regenerative medicine [6] and other fields [7]. Recently, the development of intelligent materials has prompted increasing interest in flexible and stretchable multi-functional intelligent hydrogels. However, conventional intelligent hydrogels typically possess single-response performance and limited mechanical properties, which greatly hinder their practical applications [8,9].

Specifically, for photoresponsive hydrogels, photochromic monomers, such as spiropyran (SP) [10,11,12], 4,4-bipyridine [13], azobenzene [14] and diarylethenes [15], have been introduced into hydrogels to develop optical displays. Photochromic moieties can be integrated into hydrogel structures via dynamic coordination [16], in situ co-polymerisation [17], dynamic host–guest interaction [18] and micellar co-polymerisation [19]. As an organic material with reversible photochromic properties, SP undergoes reversible intramolecular rotation between coloured and colourless isomers upon ultraviolet (UV) and visible light irradiation [20]. Moreover, under UV light exposure, the closed isomer of SP in the hydrogel network can be converted into open zwitterionic isomer merocyanine (MC) [21].

Nowadays, manufacturing high-strength hydrogels is not an arduous task [22,23]. Several types of high-strength hydrogels have been prepared, including double-network [24], nanocomposite [25], ion-cross-linked [26], hydrophobic interaction [27] and hybrid cross-linked hydrogels [28]. As a representative example of tough hydrogels, double-network hydrogels are composed of two mutually permeable and independent networks. When single-network hydrogels are subjected to external forces, energy dissipation within the hydrogel proceeds via a single pathway, resulting in weak mechanical properties. Conversely, owing to the synergistic effect of the two networks, the double-network hydrogels are effectively strengthened and toughened [29,30]. Among them, the hybrid cross-linked hydrogels typically consist of a singular network with multiple cross-linking points that increase the cross-linking density, which can be precisely adjusted for substantially enhanced mechanical properties [31,32]. Consequently, the hybrid cross-linked hydrogels based on ion coordination cross-linking have the advantages of a simplified preparation process and exceptional mechanical properties [33]. Benefitting from the dynamic reversible characteristics of ionic cross-links, the cross-linking strength can be controlled via pH or by introducing competitive ligands, which endows the hydrogels with reversible shape memory function [34].

**Figure 1:**
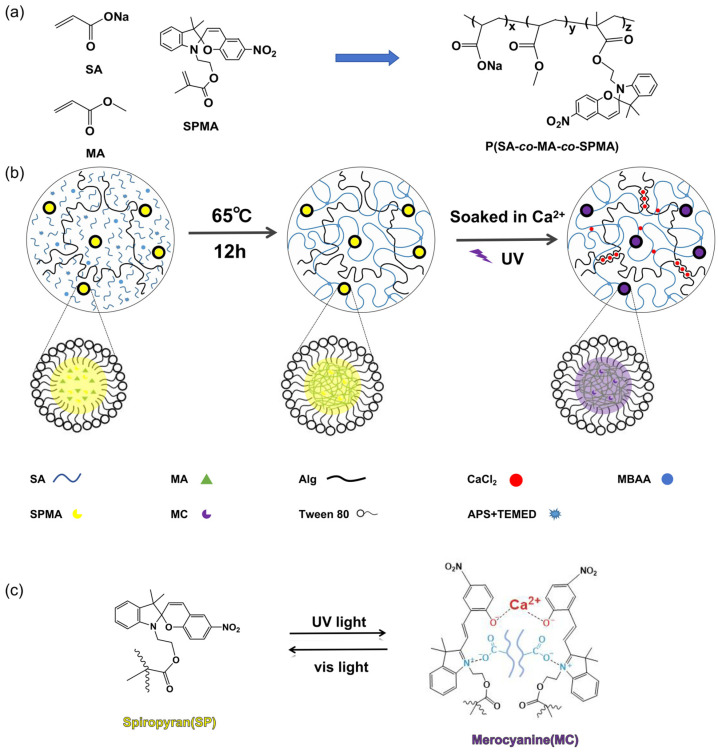
(**a**) Monomer-bonding mode in the poly(sodium acrylate-*co*-methyl acrylate-*co*-spiropyran) (P(SA-*co*-MA-*co*-SPMA)) network; (**b**) synthesis route for the hybrid cross-linked double-network photochromic hydrogel; (**c**) under the irradiation of ultraviolet (UV) and visible (vis) light, the reversible structural transition between spiropyran (SP, closed ring) and merocyanine (MC, open ring) can trigger colour change and recovery, respectively. The more polar MC can interact and coordinate with carboxylate ions and Ca^2+^ in the polymer chain (*N*,*N*′-methylenebisacrylamide (MBAA); ammonium persulfate (APS); *N*,*N*,*N*′,*N*′-tetramethylethylenediamine (TEMED)).

Herein, a photochromic hydrogel with shape memory properties and high mechanical strength was prepared by embedding acrylate derivatives of spiropyran functional groups (SPMA) into a double-network hydrogel via the micellar co-polymerisation method. Figure 1 shows the synthetic route for the ion-cross-linked hybrid double-network photochromic hydrogel using a simple one-step method. As shown in Figure 1b, natural polymer sodium alginate (Alg) was used as the first network and poly(acrylate-*co*-methyl acrylate-*co*-spiropyran) (P(SA-*co*-MA-*co*-SPMA)) was used as the second network. In addition, Ca^2+^ was introduced into the hydrogel network to undergo coordination complexation with carboxyl (–COO^−^) groups from the two molecular chains of Alg and P(SA-*co*-MA-*co*-SPMA) to obtain high-strength ion-cross-linked hybrid photochromic hydrogels [35,36]. The ionic cross-links and covalent cross-links in the system synergistically enhanced the hydrogel properties to obtain a high-strength hybrid cross-linked double-network hydrogel, which exhibited shape memory properties owing to the dynamic reversible characteristics of the ionic cross-links. In addition, as shown in Figure 1c, the photochromic structure in the hydrogel network changed from the colourless closed SP state to the purple open MC zwitterionic isomer state upon UV light irradiation. The increase in polarity led to an interaction between amphoteric MC isomer and –COO^−^ and Ca^2+^ on the molecular chain of the hydrogel, which further enhanced its mechanical properties and extended its shape recovery time. At last, these hydrogels with adjustable mechanical properties and reversible photochromic behaviour, fabricated via this simple method, are attractive candidates for optical displays and various biomedical and soft artificial intelligence systems [37].

## 2. Materials and Methods

### 2.1. Materials

Sodium alginate powder (Alg), sodium acrylate (SA) and methyl acrylate (MA) were purchased from Aladdin Industrial Corporation; *N*,*N*′-methylenebisacrylamide (MBAA), dichloromethane (CH_2_Cl_2_), α-methacrylic acid (MAA), ammonium persulfate (APS), Tween 80 and anhydrous calcium chloride (CaCl_2_) were supplied by Sinopharm Chemical Reagent Co., Ltd., Beijing, China. 4-Dimethylaminopyridine (DMAP), *N*,*N*′-dicyclohexylcarbodiimide (DCC) and *N*,*N*,*N*′,*N*′-tetramethylethylenediamine (TEMED) were purchased from Aladdin Industrial Corporation, Shanghai, China. 1-(2-Hydroxyethyl)-3,3-dimethylindolino-6′-nitrobenzopyrylospiran (SPOH) was supplied by TCI (Shanghai) Development Co., Ltd., Shanghai, China. Deionised water was used in all experiments (18.2 Ω cm resistivity at 25 °C).

### 2.2. Preparation of SPMA

The acrylate derivatives of spiropyran functional group (SPMA) monomer was synthesised via the esterification of MAA and the photochromic compound SPOH using CH_2_Cl_2_ as a solvent, DCC as a promoting agent and DMAP as a catalyst. Figure S1a shows a schematic of the synthesis process. The preparation process was as follows: SPOH (0.200 g, 0.568 mmol), MAA (0.488 g, 5.800 mmol), DMAP (0.070 g, 0.573 mmol), DCC (1.756 g, 8.510 mmol) and CH_2_Cl_2_ (100 mL) were added to a three-neck flask, which was stored in dark under nitrogen gas for the reaction to proceed at room temperature for 24 h. After a complete conversion of SPOH, the mixture was extracted with CH_2_Cl_2_, and washed 2 times with a solution of 10% aqueous HCl, 2 times with saturated NaHCO_3_ (aq) and 3 times more with deionised water. The resulting colourless liquid was separated, dried over anhydrous MgSO_4_ and filtrated. After solvent evaporation, the product was recrystallised from hexane: benzene (1:1, v:v) and vacuum-dried at 45 °C for 48 h. ^1^H NMR (400 MHz, CDCl_3_, TMS, ppm): δ = 8.03–7.99 (m, 2H, H_13_ and H_14_), 7.21 (td, 1H, H_15_), 7.10 (d, 1H, H_11_), 6.90 (dd, 2H, H_10_ and H_12_), 6.75 (d, 1H, H_8_), 6.71 (d, 1H, H_9_), 6.07 (t, 1H, H_2_), 5.87 (d, 1H, H_7_), 5.56 (t, 1H, H_2_), 4.30 (t, 2H, H_3_), 3.59–3.40 (m, 2H, H_4_), 1.92 (t, 3H, H_1_), 1.28 (s, 3H, H_5_) and 1.17 (s, 3H, H_6_) (Figure S1b) [38]. The ^1^H NMR spectrum was recorded using a Bruker AVANCE III-400 instrument (Bruker Co., Ferranden, Switzerland) at room temperature.

### 2.3. Preparation of Hybrid Cross-Linked Double-Network Photochromic Hydrogels

Firstly, photochromic monomer SPMA (0.1 mol% MA) was dissolved in hydrophobic monomer MA (50 wt% SA) to form an oil phase, and sodium Alg powder (1.6 wt% SA), SA (2.5 mol/L) and the MBAA cross-linking agent (0.4 mol% SA) were dissolved in deionised water to form an aqueous phase. Then, under the action of the Tween 80 emulsifier (1.5 wt% water), the oil phase was uniformly dispersed in the water phase using a high-speed dispersion machine to prepare a pre-polymer solution. Finally, 0.2 mol% of REDOX initiators (APS and TEMED) was added to the pre-polymer solution to form a mixed aqueous solution, which was poured into a reaction tank (100 mm × 100 mm) composed of a pair of parallel glass plates and a polyester film separated by a hollow silicone rubber gasket with a thickness of about 1 mm. The mixture was reacted in an oven at 65 °C for 12 h and then polymerised to form a sodium alginate/poly(acrylate-*co*-methyl acrylate-*co*-spiropyran) Alg/P(SA-*co*-MA-*co*-SPMA) double-network photochromic hydrogel.

The resulting hydrogels were soaked in CaCl_2_ solutions of different concentrations to introduce ionic coordination cross-linking points, affording high-strength ion-cross-linked hybrid Alg/P(SA-*co*-MA-*co*-SPMA)/Ca^2+^ photochromic hydrogels. To ensure the accuracy of the CaCl_2_ solution’s concentration during immersion, three hydrogel samples (30 mm long, 5 mm wide and 1 mm thick) were immersed in 50 mL of CaCl_2_ aqueous solution for 12 h. The corresponding samples were defined as Alg/P(SA-*co*-MA-*co*-SPMA)/Ca^2+^_XM_ and Alg/P(SA-*co*-MA-*co*-SPMA)/Ca^2+^@UV, where X is the concentration of Ca^2+^ in the impregnation, and UV represents that the hydrogel sample was irradiated with UV light after being impregnated in Ca^2+^ solution. P(SA-*co*-MA-*co*-SPMA)/Ca^2+^ hydrogels were prepared using the same reagent concentration and preparation method as described above but without the sodium Alg network.

### 2.4. Measurements

#### 2.4.1. Tensile Measurement

At room temperature, uniaxial tensile tests were conducted using an electronic universal tensile testing machine (CMT6103, MTS Co., Shanghai, China) on rectangular hydrogel samples at a constant tensile speed of 50 mm min^−1^. The sensor was set to 1 kN. Unless otherwise specified, three measurements were performed for each sample. The elastic modulus (*E*) was calculated from the slope (5–10%) of the initial linear region of the stress–strain curve. The work of tension was determined by integrating the area under the stress–strain curve until the sample broke. The nominal stress (*σ*) was calculated from the tensile force and the initial cross-sectional area of the sample as follows:

(1) σ=F/bd

where *F* is the maximum load of the sample, *b* is the sample width and *d* is the sample thickness.

The original gauge length was defined as the distance between the upper and lower clamps of the sample before stretching. The gauge length at the time of fracture was defined as the distance between the clamps at the time of fracture after the sample was continuously stretched. The elongation at break (*ε*) was calculated as follows:

(2) $$\varepsilon =(L-L_{0})/L_{0}$$

where *L*_0_ is the original gauge length of the sample and *L* is the gauge length when the sample breaks.

#### 2.4.2. Fourier Transform Infrared Spectroscopy

The hydrogel was placed in a vacuum oven at 65 °C for 12 h to dehydrate the sample. The obtained solid sample was mixed with an appropriate amount of potassium bromide powder to form a uniform powder and dried in a vacuum oven at 80 °C for 3 h. The Fourier transform infrared (FTIR) spectra were recorded on a Bruker TENSOR 27 FTIR spectrometer (Bruker Co., Karlsruhe, Germany). All the spectra were obtained with 16 scans and a resolution of 4 cm^−1^ in the range 4000–400 cm^−1^. All the spectra were obtained at ambient temperature (22–25 °C).

#### 2.4.3. UV-Vis Spectroscopy

A Hitachi U-3900 UV-vis spectrophotometer (Hitachi Co., Tokyo, Japan) was used to measure the absorbance with a scanning speed of 300 nm/min. All photochromic hydrogel samples were treated with UV light using a UV-curable machine (a mercury lamp with a maximum at 365 nm and an intensity of 5.3 mW cm^−2^, ELC-500, Electro-Lite Co., Tokyo, Japan). Then, the sample was irradiated with UV light for different durations and the corresponding UV-vis spectra were collected. After the sample was irradiated with UV light for 30 s, the UV-vis spectra were collected after visible light irradiation for different times using a wight light LED lamp (OPPLE, Shanghai, China, 55 W) with an intensity of 0.8 mW cm^−2^.

#### 2.4.4. Scanning Electron Microscopy Imaging

The cross-sectional morphology of the hydrogel samples was observed using a Hitachi SU8010 field emission scanning electron microscope (SEM) (Hitachi Co., Tokyo, Japan.). In detail, samples with small notches were frozen and brittle in liquid nitrogen, and the frozen hydrogels were placed in a freeze dryer for 48 h to dehydrate them into aerohydrogels. Then, the fractured surfaces of the samples were gold-coated using a JUC-500 magnetron-sputtering device (JEOL Co., Tokyo, Japan.) and observed with an accelerating voltage of 5 kV.

#### 2.4.5. Water Content Measurement

The water content of the hydrogels was measured by using a Shimadzu MOC-120H moisture balance (Shimadzu Co., Kyoto, Japan). Specifically, the hydrogels were wiped to remove the water from their surface and then dried at 110 °C to obtain completely dry samples. The water content (wt%) is defined as the percentage of the weight of water in the hydrogel and the total weight of the hydrogel.

#### 2.4.6. The Shape Recovery Rate

The Alg/P(SA-*co*-MA-*co*-SPMA) hydrogel was cut into straight strips (70 mm long, 10 mm wide and 1 mm thick) and soaked in 2.8 M Ca^2+^ solution for 4 h, which was used as a ion-cross-linked agent to fix the original shape of the Alg/P(SA-*co*-MA-*co*-SPMA)/Ca^2+^ hydrogel. The strips were put into pure water at room temperature for 120 s, and the hydrogel softened owing to the blocking of ionic cross-links. The straight hydrogel strip was bent to form a circle and soaked in 2.8 M Ca^2+^ solution for 4 h to form the temporary shape of the hydrogel strip, and we denoted the bending angle of this circle as *θ*_m_. Subsequently, the hydrogel circle was immersed in pure water to initiate shape recovery, and the deformation angle after immersing in pure water for 60 s was defined as *θ*_r_. The shape recovery rate (*R*_r_) is defined by the following equation:

(3) $$R_{r}=\theta_{r}/\theta_{m}\times \;100\%$$

## 3. Results and Discussion

### 3.1. FTIR Spectroscopy and UV–Vis Spectroscopy

**Figure 2:**
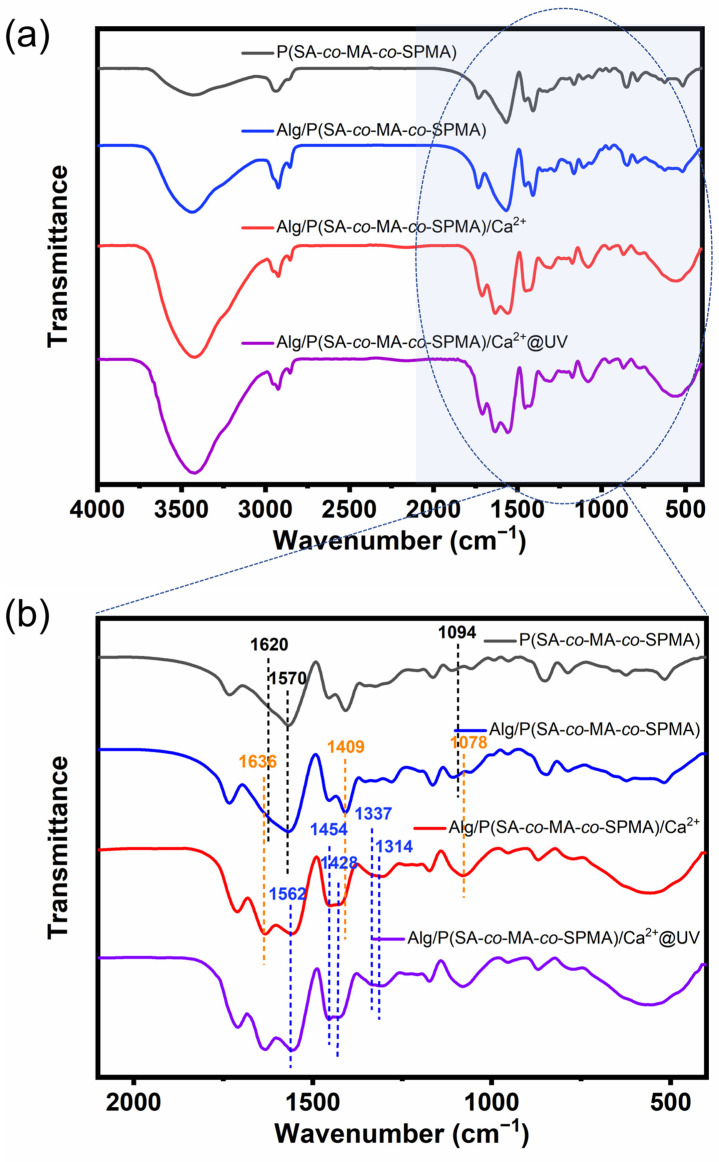
(**a**) FTIR spectra of hydrogels P(SA*-co*-MA-*co*-SPMA), Alg/P(SA-*co*-MA-*co*-SPMA), Alg/P(SA-*co*-MA-*co*-SPMA)/Ca^2+^_2.8M_ and Alg/P(SA-*co*-MA-*co*-SPMA)/Ca^2+^_2.8M_@UV; (**b**) FTIR spectra of hydrogels in the range 2100–400 cm^−1^.

FTIR spectroscopy was performed to analyse the chemical structures of the hydrogels, as shown in Figure 2. A comparison of the FTIR spectra of the poly(acrylate-*co*-methyl acrylate-*co*-spiropyran) (P(SA-*co*-MA-*co*-SPMA)) and alginate/poly(acrylate-*co*-methyl acrylate-*co*-spiropyran) (Alg/P(SA-*co*-MA-*co*-SPMA)) hydrogels reveals the appearance of C=O symmetric stretching vibration and C–O stretching vibration bands of Alg at 1620 and 1094 cm^−1^, respectively, indicating the successful incorporation of Alg into the P(SA-*co*-MA-*co*-SPMA) hydrogel network [39]. The carboxyl group’s asymmetric stretching vibrations shifted from 1570 cm^−1^ in the spectrum of P(SA-*co*-MA-*co*-SPMA) hydrogel to 1563 cm^−1^ in the spectra of Alg/P(SA-*co*-MA-*co*-SPMA) hydrogel, which indicates the formation of hydrogen bonds between the carboxyl group and hydroxyl group. Moreover, the C–O stretching vibration band at 1078 cm^−1^ and the C=O stretching vibration band at 1636 cm^−1^ in the FTIR spectra of the Alg/P(SA-*co*-MA-*co*-SPMA)/Ca^2+^ hydrogel were enhanced compared with those of Alg/P(SA-*co*-MA-*co*-SPMA), whereas the –COOH stretching vibration band at 1409 cm^−1^ was weakened. These results imply that the added Ca^2+^ ions formed coordination complexes with the Alg/PSA molecular chains [40]. Finally, the –C–O– stretching vibration band at 1428 cm^−1^ and the –C–N^+^ stretching vibration band at 1314 cm^−1^ of the Alg/P(SA-*co*-MA-*co*-SPMA)/Ca^2+^ hydrogel were enhanced after UV exposure, which indicates the photoinduced conversion of SP into MC in the polymer [41,42]. Upon UV irradiation, the stretching vibration band owing to tertiary C–N shifted to a higher energy from 1327 to 1335 cm^−1^, whereas the –COO^−^ stretching vibration bands at 1562 and 1454 cm^−1^ were enhanced, potentially owing to the interaction between the zwitterionic MC structure and Ca^2+^ or –COO^−^ in the network. Figure S2 shows the FTIR spectra of photochromic monomer SPMA. The band at 2963 cm^−1^ is attributed to the stretching vibration of v(C–H) in –CH_3_ (asymmetric), and the band at 2922 cm^−1^ is attributed to v(C–H) in –CH_2_– (asymmetric). The –CH_3_ asymmetric stretching vibration band at 2963 cm^−1^ in the FTIR spectra of SPMA are stronger compared with those of SPOH. Furthermore, it an unsaturated ester C=O vibration band at 1718 cm^−1^ and a C=C stretching vibration band at 1650 cm^−1^ were found in the FTIR spectra of SPMA. These results indicated that SPMA was synthesised successfully.

**Figure 3:**
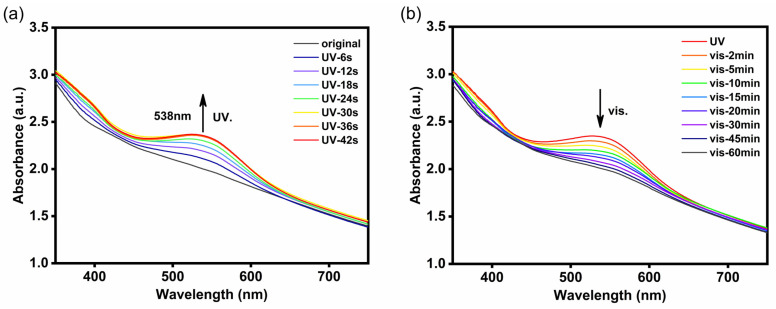
(**a**) UV-vis absorption spectra of Alg/P(SA-*co*-MA-*co*-SPMA)/Ca^2+^_2.8M_ hydrogel upon UV light irradiation at different times; (**b**) UV-vis absorption spectra of Alg/P(SA-*co*-MA-*co*-SPMA)/Ca^2+^_2.8M_@UV hydrogel during the visible-light irradiation process.

Subsequently, the photochromic behaviour of hydrogels was measured using the UV–vis spectrophotometer. Taking Alg/P(SA-*co*-MA-*co*-SPMA)/Ca^2+^_2.8M_ hydrogel as an example (Figure 3a), the hydrogel could become photochromic in 6 s under UV light irradiation, indicating an extremely rapid photochromic response speed. The UV-vis spectrum of Alg/P(SA-*co*-MA-*co*-SPMA)/Ca^2+^_2.8M_ displayed obvious SP open-loop characteristic peaks at 538 nm, which reached saturation after 30 s of irradiation. As shown in Figure 3b, the fading behaviour of Alg/P(SA-*co*-MA-*co*-SPMA)/Ca^2+^_2.8M_@UV hydrogels was measured through the UV-vis spectrophotometer. The SP open-loop characteristic peaks at 538 nm gradually weakened during visible-light irradiation process, and the hydrogels faded from a coloured state to a colourless state after 1 h. This indicates that the hydrogels have the ability to retain their colour for a certain period of time.

### 3.2. Mechanical Properties of the Hydrogels

To study the effect of the first-network sodium alginate (Alg) on the mechanical properties of P(SA-*co*-MA-*co*-SPMA) hydrogels, tensile properties were investigated. As shown in Figure S3a, the tensile strength of the P(SA-*co*-MA-*co*-SPMA) hydrogels was less than 23 kPa with a maximum elongation at break of 220%. After the addition of Alg, the tensile strength and maximum elongation at break of the hydrogels greatly increased to 33.8 kPa and nearly 500%, respectively, because many long-chain Alg molecules entangled with the P(SA-*co*-MA-*co*-SPMA) molecular chains in the covalently cross-linked network. Simultaneously, hydrogen bonding was formed between the –COO^−^ groups in the P(SA-*co*-MA-*co*-SPMA) chains and hydroxyl groups in the Alg chains, obviously increasing the tightness of the whole hydrogel network. In such a network, the stress can be well dispersed when external forces are applied to the Alg/P(SA-*co*-MA-*co*-SPMA) hydrogel, thus increasing its tensile strength and elongation at break.

**Figure 4:**
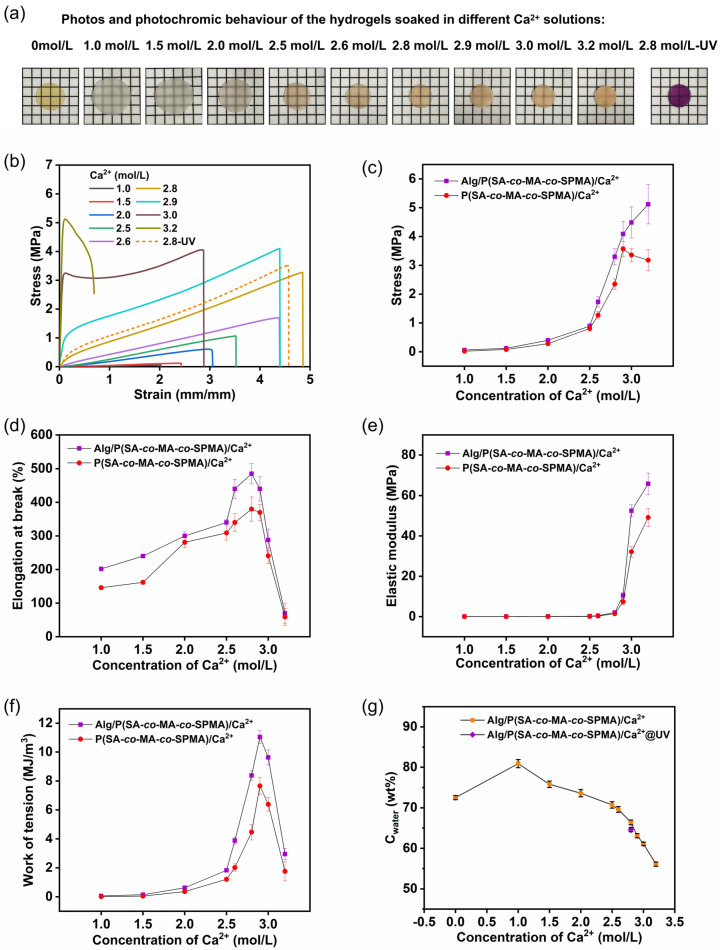
The effect of the Ca^2+^ concentration of the soaking solution and UV irradiation on the mechanical properties and morphology of the hydrogels. (**a**) Photos of Alg/P(SA-*co*-MA-*co*-SPMA)/Ca^2+^ hydrogels soaked in different Ca^2+^ solutions, and photochromic behaviour of Alg/P(SA-*co*-MA-*co*-SPMA)/Ca^2+^_2.8M_@UV; (**b**) stress–strain curves of Alg/P(SA-*co*-MA-*co*-SPMA)/Ca^2+^ hydrogels soaked in different Ca^2+^ solutions. Comparison of the mechanical properties of Alg/P(SA-*co*-MA-*co*-SPMA)/Ca^2+^ double-network hydrogels and P(SA-*co*-MA-*co*-SPMA)/Ca^2+^ hydrogels. (**c**) Tensile strength; (**d**) elongation at break; (**e**) elastic modulus; (**f**) work of tension. (**g**) Water content (*C*_water_) of photochromic Alg/P(SA-*co*-MA-*co*-SPMA)/Ca^2+^_2.8M_@UV and Alg/P(SA-*co*-MA-*co*-SPMA)/Ca^2+^ hydrogels in different Ca^2+^ solutions.

These results demonstrate that weak Alg/P(SA-*co*-MA-*co*-SPMA) hydrogels were converted into strong and tough hydrogels by forming Ca^2+^–COO^−^ coordination complexes that acted as additional cross-links to strengthen the hydrogel matrix. When the hydrogel was soaked in 2.8 M Ca^2+^ solutions, its elongation at break reached maximum values of 490%, a tensile strength of 3.3 MPa, an elastic modulus of 2.0 MPa and a work of tension of 8.4 MJ m^−3^ (Figure 4b and Table 1). Compared with those of the hydrogel without Ca^2+^, the tensile strength and the work of tension increased by 97 and 98 times, respectively. This is because soaking in Ca^2+^ solutions produces not only more molecular chain entanglements but also cross-links owing to the formation of complexes between Ca^2+^ and –COO^−^. Control hydrogels without Alg exhibited similar mechanical behaviour to that of the Alg/P(SA-*co*-MA-*co*-SPMA)/Ca^2+^ double-network hydrogels. A comparison of the mechanical properties of the P(SA-*co*-MA-*co*-SPMA)/Ca^2+^ hydrogels and Alg/P(SA-*co*-MA-*co*-SPMA)/Ca^2+^ double-network hydrogels is shown in Table 1. Compared with the P(SA-*co*-MA-*co*-SPMA)/Ca^2+^ hydrogels, the Alg/P(SA-*co*-MA-*co*-SPMA)/Ca^2+^ double-network hydrogels have a higher tensile strength and initial elastic modulus, indicating that the first Alg network helps improve the mechanical properties of the hydrogel. This can be attributed to Alg increasing the entanglement of the hydrogel network, which results in the formation of egg-box-like ionic interactions between Alg and Ca^2+^ as the strong dynamic bonds, leading to more intermolecular interactions in the hydrogel networks. The SEM images of the P(SA-*co*-MA-*co*-SPMA) and Alg/P(SA-*co*-MA-*co*-SPMA) hydrogel clearly display a multi-scale network structure containing abundant micropores with an average size of 1 µm (Figure S4b). After soaking in Ca^2+^, no clear network structure can be observed in the SEM images of the Alg/P(SA-*co*-MA-*co*-SPMA)/Ca^2+^_2.8M_ hydrogel, which contains many micelle microspheres with an average size of 1.5 µm (Figure S4e). This is because the hydrogel network becomes dense after being soaked in 2.8 M Ca^2+^, which leads to a volume shrinkage and transparency decrease. We compared the tensile strength and elongation at break of Alg/P(SA-*co*-MA-*co*-SPMA)/Ca^2+^ DN hydrogel with those in our previous work [40]. When the single hydrogel was soaked in 2.8 M Ca^2+^ solutions, the tensile strength and elongation at break of the hydrogel reached a tensile strength of 2.6 MPa and an elongation at break of 458%. However, in this work, the DN hydrogel exhibited a tensile strength of up to 3.3 MPa and a higher elongation at break of up to about 500%. The high strength of DN hydrogel originated from the wide distribution of bonding strength of coordination complexes as well as the covalent bonding between the cross-link network; the strong dynamic bonds and covalent bonds served as permanent cross-links to maintain the integrity of hydrogels, whereas the relatively weak dynamic bonds and hydrogen bonds behaved as reversible and sacrificial bonds to dissipate energy.

To study the effect of the Ca^2+^ concentration of the soaking solution on the mechanical properties of the Alg/P(SA-*co*-MA-*co*-SPMA)/Ca^2+^ hydrogels, the tensile properties were investigated (Figure 4b and Table 1). The tensile strength of the Alg/P(SA-*co*-MA-*co*-SPMA)/Ca^2+^ hydrogel soaked in solutions with the Ca^2+^ concentrations ranging from 1.0 to 3.2 M gradually increased from 0.061 to 5.1 MPa (Figure 4c). With the continuous increase in the Ca^2+^ concentration, the concentration of the external environment solution exceeded that of the hydrogel internal environment, causing a shrinkage and decrease in the water content of the hydrogel (Figure 4g). Optical photos of the hydrogels are shown in Figure 4a. With the volume shrinkage of the hydrogel, more ionic cross-links and more entanglements were formed between the molecular chains of the hydrogel network, which were tightly clustered and have small porosity. The work of tension of the Alg/P(SA-*co*-MA-*co*-SPMA)/Ca^2+^ hydrogel also gradually increased from 0.056 to 11 MJ m^−3^ with increasing Ca^2+^ concentrations from 1.0 to 2.9 M because the metal coordination cross-links were cleaved under external stress, facilitating energy dissipation (Figure 4f). The elongation at break increased first and then decreased with increasing Ca^2+^ concentrations, reaching the maximum value of 490% at a Ca^2+^ concentration of 2.8 M (Figure 4d). With the increase in Ca^2+^ concentration, the elastic modulus of the hydrogel are significantly improved (Figure 4e). As shown in the images (Figure S4b), the Alg/P(SA-*co*-MA-*co*-SPMA)/Ca^2+^_2.0M_ hydrogel had a bigger pore size than the Alg/P(SA-*co*-MA-*co*-SPMA) hydrogel did. As the concentration of Ca^2+^ increased, the network structure of hydrogels shrunk and such pores’ disappearance was visualised. Scanning electron microscopy (SEM) images of Alg/P(SA-*co*-MA-*co*-SPMA)/Ca^2+^_2.6M_ hydrogel clearly display many microspheres (Figure S4d). When Ca^2+^ increases, the microspheres decrease significantly in images of Alg/P(SA-*co*-MA-*co*-SPMA)/Ca^2+^_3.0M_ hydrogel. This is because the hydrogel network becomes denser after being soaked in 3.0 M Ca^2+^, which leads to the polymer network shrinking and tightly wrapping the microspheres. Upon increasing the Ca^2+^ concentration, the osmotic pressure inside and outside the hydrogel also increase, driving hydrogel shrinkage and more Ca^2+^ ions to penetrate into the hydrogel, forming more coordination structures with the –COO^−^ groups on the macromolecular chains and making the cross-linked network more compact, thus increasing the tensile strength and elastic modulus of the hydrogel. When the concentration of Ca^2+^ ions further increases within the range of 2.9–3.2 M, the volume of the hydrogel obviously shrinks, increasing the density of the macromolecular chains of the hydrogel. Ca^2+^ ions form excess ionic cross-links structures with the –COO^−^ groups in the macromolecular chains, restricting the free movement of the macromolecular chain segments and in turn decreasing the elongation at break decrease to a certain extent.

After 30 s of UV irradiation, the tensile strength of the Alg/P(SA-*co*-MA-*co*-SPMA)/Ca^2+^_2.8M_ hydrogel increased by 9.4% from 3.2 to 3.5 MPa (Figure 4c and Table 1), which is due to the presence of a large number of polar zwitterionic MC structures in the network. At this point, simultaneously with the volume shrinkage [43], the water content of the hydrogel decreased from 66.4% to 64.6% (Figure 4g). The zwitterionic MC structures can also interact with Ca^2+^, increasing the physical interactions in the co-polymerisation network, further enhancing the mechanical properties of the hydrogel. This result is consistent with that of FTIR spectral analysis. The hydrogels also exhibited excellent photoisomerisation reversibility and fatigue resistance at room temperature. The fatigue resistance to light was investigated by subjecting the same hydrogel to 10 cycles of UV-vis irradiation (Figure S5). During the first six colouration/decolouration cycles, the intensity at *λ*_max_ decreased by 5%. However, during the next four cycles, the intensity at *λ*_max_ only decreased by another 5%. With the increasing number of cycles, the degree of reduction in absorption intensity decreased. These results show that metal coordination can substantially improve the mechanical properties of the hydrogels, which can be controllably adjusted within a certain range. The introduction of metal coordination in photochromic hydrogels can be enhanced via UV irradiation, resulting in excellent light fatigue resistance.

### 3.3. Shape Memory of the Hydrogels

**Figure 5:**
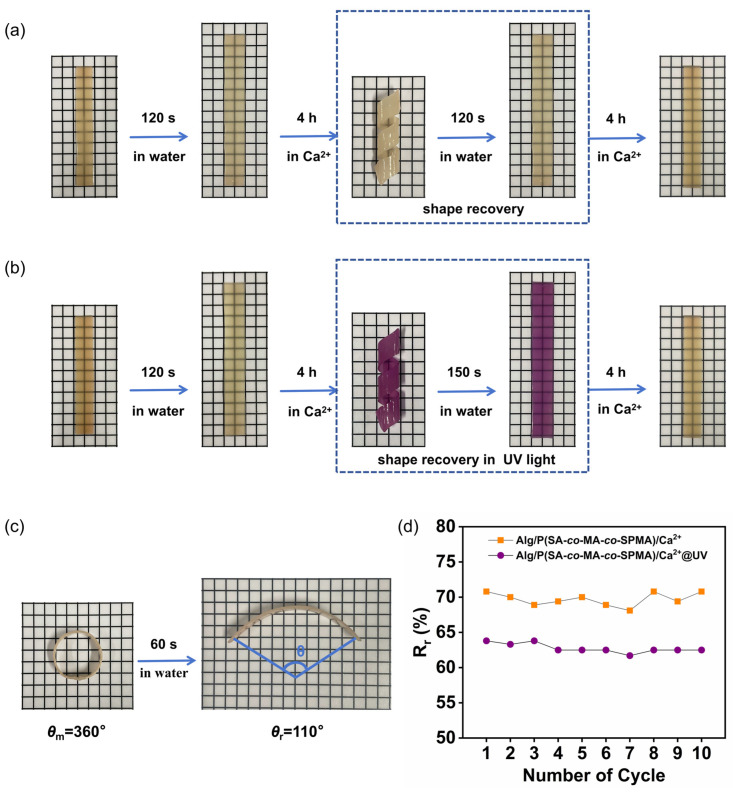
(**a**) Schematic of the shape memory behaviour of the Alg/P(SA-*co*-MA-*co*-SPMA)/Ca^2+^_2.8M_ hydrogel mediated by pure-water switching; (**b**) schematic of the shape memory behaviour of the Alg/P(SA-*co*-MA-*co*-SPMA)/Ca^2+^_2.8M_ hydrogel mediated by pure-water switching under UV light; (**c**) schematic of the shape recovery behaviour of the Alg/P(SA-*co*-MA-*co*-SPMA)/Ca^2+^_2.8M_ hydrogel forming a circle shape upon immersion in pure water for 60 s; (**d**) shape recovery rate (*R*_r_) after 10 cycles of shape fixation and relaxation of the same hydrogel sample exposed to visible light and ultraviolet light.

As expected, the dynamic ionic coordination bonds endowed the hydrogels with shape memory functionalities [44]. The stability of coordination complexes is influenced by the Ca^2+^ concentration, indicating their dynamic nature. Because of the reversibility of coordination bonds of the system of P(SA-*co*-MA-*co*-SPMA) and Ca^2+^, the shape fixation and recovery of hydrogels can be realised by switching the Ca^2+^ concentration in the hydrogels. When the hydrogel is soaked in Ca^2+^ solution, the hydrogel shrinkage and Ca^2+^ ions penetrate into the hydrogel, forming coordination structures with the –COO^−^ groups on the macromolecular chains and programming a temporary shape. Moreover, the ion-cross-linked hydrogels usually produce a reversible shape memory effect by controlling the pH value or introducing competitive ligands [34]; however, the Alg/P(SA-*co*-MA-*co*-SPMA)/Ca^2+^_2.8M_ hydrogel can quickly recover its shape in pure water. As shown in Figure 5a, an Alg/P(SA-*co*-MA-*co*-SPMA)/Ca^2+^_2.8M_ hydrogel sample was put into pure water at room temperature for 120 s, and the hydrogel softened owing to the blocking of ionic cross-links. This softening indicates that the dynamic cross-links between P(SA-*co*-MA-*co*-SPMA) and Ca^2+^ were damaged, with the egg-box-like ionic interactions between Alg and Ca^2+^ and the covalent cross-links in the P(SA-*co*-MA-*co*-SPMA) network acting as permanent netpoints. After soaking the softened hydrogel in 2.8 M Ca^2+^ solution for 4 h to form and consolidate a spiral shape, the hydrogel maintained the spiral shape after removing the external force, achieving shape programming. Figure 5a shows the shape memory property of the hydrogel upon immersion in pure water and Ca^2+^ solution. The Alg/P(SA-*co*-MA-*co*-SPMA)/Ca^2+^_2.8M_ hydrogel can recover its original flat shape in only 120 s in pure water and in 150 s in the UV curing box (Figure 5b). When the hydrogel is immersed in pure water, the osmotic pressure outside the hydrogels decreases, quickly driving hydrogel swelling and Ca^2+^ ions to penetrate into pure water, destructing coordination structures with the –COO^−^ groups on the macromolecular chains and driving the hydrogel to recover shape. The longer time required for the hydrogel to complete shape recovery under UV light is due to the presence of abundant polar zwitterionic MC structures in the network after UV irradiation. The polarity increase may promote the interaction between the MC isomers and –COO^−^ groups in the molecular chains of the hydrogel. Meanwhile, the zwitterionic MC structures can also interact with Ca^2+^, increasing the physical entanglement in the co-polymerisation network, making the cross-linked network more compact. After recovering the original flat shape in pure water, the hydrogel can form a new shape through being soaked in 2.8 M Ca^2+^ solution, achieving shape programming again.

In practical applications of hydrogels, the reversibility of shape fixation and relaxation is very important. As shown in Figure 4c, a softened hydrogel soaked in 2.8 M Ca^2+^ solution for 4 h to consolidate its shape forms a circle with a deformation angle, *θ*_m_, after shape memory. Subsequently, the hydrogel can recover its shape after immersion in pure water for 60 s, and the deformation angle after shape recovery is *θ*_r_. The shape recovery rate (*R*_r_) can be calculated using the ratio between *θ*_r_ and *θ*_m_. As shown in Figures S6 and S7, reversibility was investigated by subjecting the same hydrogel to 10 cycles of shape fixation and relaxation and separately recording the *θ*_m_ and *θ*_r_ values. As shown in Figure 4d, with the increasing number of cycles, *R*_r_ exhibits excellent repeatability, indicating the excellent reversibility of the shape fixation and relaxation of the hydrogel before and after UV irradiation.

## 4. Conclusions

Photochromic hydrogels with adjustable mechanical properties, photoreversible stability and shape memory properties with fast response were prepared using a simple one-pot method. The construction of an ion-cross-linked hybrid Alg/P(SA-*co*-MA-*co*-SPMA)/Ca^2+^ double network greatly improved the mechanical properties of the hydrogels. The mechanical properties of these materials could be adjusted through dynamic interactions between Ca^2+^ ions and –COO^−^ groups in the system. The dynamic nature of the interactions between Ca^2+^ ions and –COO^−^ groups in P(SA-*co*-MA-*co*-SPMA) network endowed the hydrogel with shape memory behaviour in pure water and excellent reversibility of shape fixation and relaxation. Furthermore, the SP structure in the hydrogel network could be controlled via UV irradiation, which considerably improved the tensile strength of the hydrogel through interactions between Ca^2+^ ions and zwitterionic MC structures. Therefore, these ion-cross-linked hybrid hydrogels with multi-functionalities are attractive candidates for optical displays and various biomedical and soft artificial intelligence systems.

## Figures and Tables

**Table 1:** Comparison of mechanical properties for Alg/P(SA-*co*-MA-*co*-SPMA)/Ca^2+^ double-network hydrogels and P(SA-*co*-MA-*co*-SPMA)/Ca^2+^ hydrogels.

*C*Ca^2+^/M	Alg/P(SA-*co*-MA-*co*-SPMA)/Ca^2+^				P(SA-*co*-MA-*co*-SPMA)/Ca^2+^			
	Tensile Strength [MPa]	Elongation at Break [%]	Elastic Modulus [MPa]	Work of Tension [MJ m^−3^]	Tensile Strength [MPa]	Elongation at Break [%]	Elastic Modulus [MPa]	Work of Tension [MJ m^−3^]
0.0 ^1^	(3.3 ± 0.8) × 10^−2^	(4.9 ± 0.4) × 10^2^	(1.2 ± 0.1) × 10^−2^	(8.6 ± 1.1) × 10^−2^	(2.2 ± 0.5) × 10^−2^	(2.2 ± 0.1) × 10^2^	(2.1 ± 0.2) × 10^−2^	(3.0 ± 0.6) × 10^−2^
1.0	(6.1 ± 1.0) × 10^−2^	(2.0 ± 0.1) × 10^2^	(3.2 ± 0.3) × 10^−2^	(5.6 ± 0.6) × 10^−2^	(2.2 ± 0.3) × 10^−2^	(1.5 ± 0.1) × 10^2^	(1.4 ± 0.3) × 10^−2^	(1.4 ± 0.5) × 10^−2^
1.5	(1.2 ± 0.2) × 10^−1^	(2.4 ± 0.1) × 10^2^	(3.9 ± 0.3) × 10^−2^	(1.5 ± 0.1) × 10^−1^	(7.5 ± 0.9) × 10^−2^	(1.6 ± 0.1) × 10^2^	(2.8 ± 0.3) × 10^−2^	(4.8 ± 0.9) × 10^−2^
2.0	(4.0 ± 0.4) × 10^−1^	(3.0 ± 0.1) × 10^2^	(8.9 ± 0.7) × 10^−2^	(6.2 ± 0.5) × 10^−1^	(2.8 ± 0.4) × 10^−1^	(2.8 ± 0.2) × 10^2^	(3.9 ± 0.5) × 10^−2^	(3.6 ± 0.5) × 10^−1^
2.5	(8.9 ± 1.1) × 10^−1^	(3.4 ± 0.1) × 10^2^	(2.2 ± 0.2) × 10^−1^	1.8 ± 0.1	(8.1 ± 0.9) × 10^−1^	(3.1 ± 0.2) × 10^2^	(1.2 ± 0.2) × 10^−1^	1.2 ± 0.2
2.6	1.7 ± 0.2	(4.4 ± 0.3) × 10^2^	(4.8 ± 0.3) × 10^−1^	3.9 ± 0.2	1.3 ± 0.1	(3.4 ± 0.3) × 10^2^	(2.8 ± 0.3) × 10^−1^	2.0 ± 0.3
2.8	3.3 ± 0.3	(4.9 ± 0.3) × 10^2^	2.0 ± 0.2	8.4 ± 0.3	2.4 ± 0.2	(3.8 ± 0.6) × 10^2^	1.5 ± 0.2	4.5 ± 0.5
2.8 ^2^	3.5 ± 0.3	(4.6 ± 0.3)) × 10^2^	2.6 ± 0.2	8.6 ± 0.3	/	/	/	/
2.9	4.1 ± 0.4	(4.4 ± 0.4) × 10^2^	(1.1 ± 0.2) × 10^1^	(1.1 ± 0.1) × 10^1^	3.6 ± 0.3	(3.7 ± 0.6) × 10^2^	7.4 ± 1.6	7.7 ± 0.6
3.0	4.1 ± 0.6	(2.9 ± 0.4) × 10^2^	(5.3 ± 0.3) × 10^1^	9.6 ± 0.5	3.4 ± 0.2	(2.4 ± 0.4) × 10^2^	(3.2 ± 0.3) × 10^1^	6.4 ± 0.5
3.2	5.1 ± 0.9	(7.0 ± 3.0) × 10^1^	(6.6 ± 0.5) × 10^1^	3.0 ± 0.4	3.2 ± 0.4	(5.7 ± 2.5) × 10^1^	(4.9 ± 0.5) × 10^1^	1.8 ± 0.7

^1^ Represents the hydrogels were not soaked in Ca^2+^ solutions. ^2^ Represents the hydrogels after UV irradiation.

## Data Availability

Data are contained within the article.

## Supplementary Materials

The following supporting information can be downloaded at https://www.mdpi.com/article/10.3390/polym16081031/s1: Figure S1: (a) The synthetic route of SPMA. (b) ^1^H NMR spectrum of SPMA monomer (solvent CDCl_3_, 400MHz). Figure S2: FTIR spectra of SPMA, SPOH and α-methacrylic acid. Figure S3: (a) Stress–strain curve of P(SA-*co*-MA-*co*-SPMA) hydrogel and Alg/P(SA-*co*-MA-*co*-SPMA) hydrogel. (b) Stress–strain curve of Alg/P(SA-*co*-MA-*co*-SPMA)/Ca^2+^ hydrogels soaked in solutions at different Ca^2+^ concentrations. Figure S4: SEM images of cross-sectional morphology of hydrogels (scale bar = 2 µm). (a) P(SA-*co*-MA-*co*-SPMA) hydrogel. (b) Alg/P(SA-*co*-MA-*co*-SPMA) hydrogel. (c) Alg/P(SA-*co*-MA-*co*-SPMA)/Ca^2+^_2.0M_ hydrogel. (d) Alg/P(SA-*co*-MA-*co*-SPMA)/Ca^2+^_2.6M_ hydrogel. (e) Alg/P(SA-*co*-MA-*co*-SPMA)/Ca^2+^_2.8M_ hydrogel. (f) Alg/P(SA-*co*-MA-*co*-SPMA)/Ca^2+^_3.0M_ hydrogel. Figure S5: Changes in A_n_/A_1_ during repeated colouration/discolourisation cycle of Alg/P(SA-*co*-MA-*co*-SPMA)/Ca^2+^_2.8M_ hydrogel irradiated by alternative UV/vis light, where A_1_ and A_n_ represent the intensities for the first and nth cycles at the maximum absorption wavelength. Figure S6: The photos of Alg/P(SA-*co*-MA-*co*-SPMA)/Ca^2+^_2.8M_ hydrogel undergoing 10 cycles of shape fixation and shape recovery. Figure S7: The photos of Alg/P(SA-*co*-MA-*co*-SPMA)/Ca^2+^_2.8M_ hydrogel undergoing 10 cycles of shape fixation and shape recovery under UV irradiation.
