# Supplementary material for: Ion-Cross-Linked Hybrid Photochromic Hydrogels with Enhanced Mechanical Properties and Shape Memory Behaviour
Source: Polymers (Basel). 2024 Apr 10;16(8):1031. doi: 10.3390/polym16081031 (PMC11054056; doi:10.3390/polym16081031)
Supplement: Supplementary file 1 [file polymers-16-01031-s001.zip › polymers-2910408-supplementary.pdf]

Supporting Information for

Ion-Cross-Linked Hybrid Photochromic Hydrogels with Enhanced Mechanical Properties and Shape Memory Behaviour

Shijun Long [1,2,3], Fan Chen [1], Han Ren [1], Yali Hu [1], Chao Chen [4,*], Yiwan Huang [1,2] and Xuefeng Li [1,2,3,*]

[1] *Hubei Provincial Key Laboratory of Green Materials for Light Industry, Hubei University of Technology, Wuhan 430068, China; longshijun.hp@163.com (S.L.); chenfan202301@163.com (F.C.); renhann@126.com (H.R.); hu_yali1@163.com (Y.H.); yiwanhuang@hbut.edu.cn (Y.H.)*

[2] *Hubei Longzhong Laboratory, Xiangyang 441000, China*

[3] *New Materials and Green Manufacturing Talent Introduction and Innovation Demonstration Base, Hubei University of Technology, Wuhan 430068, China*

[4] *Hubei Key Laboratory of Polymer Materials, Hubei University, Wuhan 430062, China*

[*]Correspondence: chenchao@hubu.edu.cn (C.C.); li_xf@mail.hbut.edu.cn (X.L.)

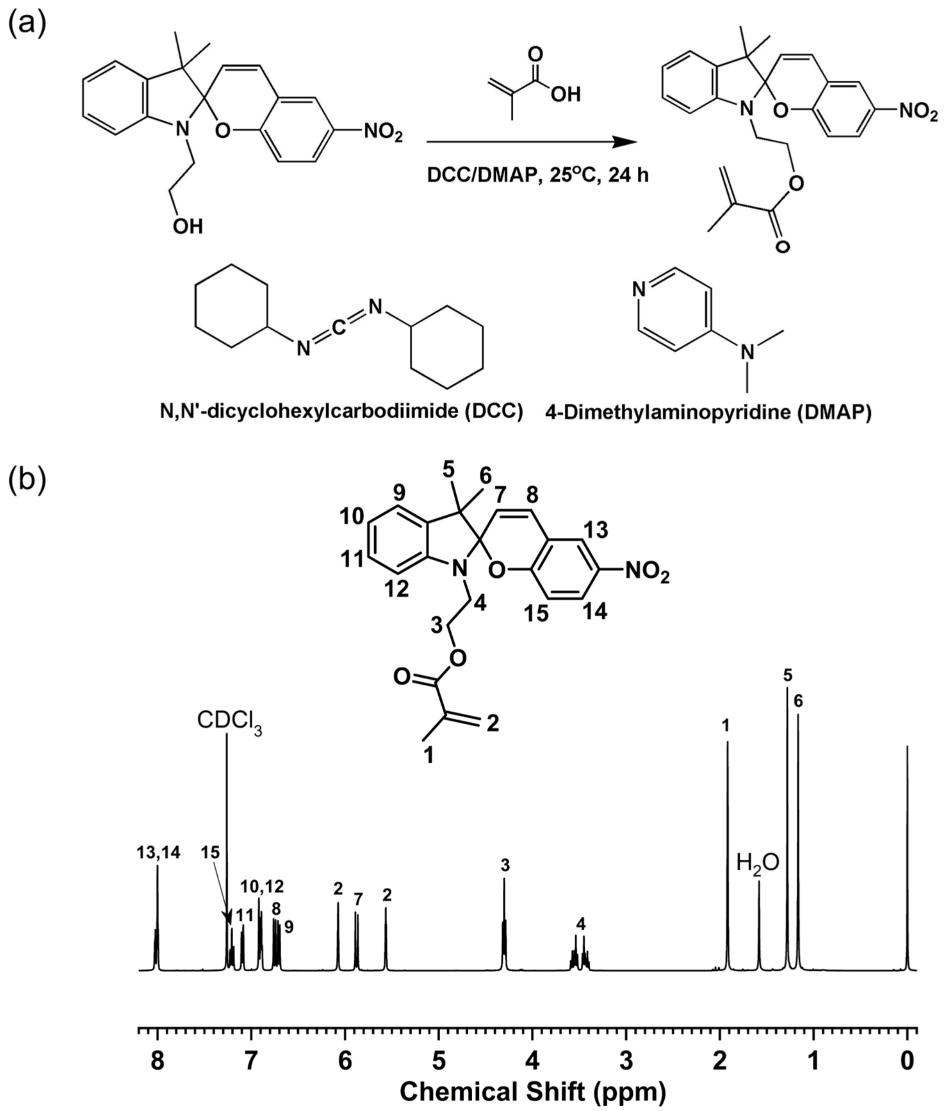

Figure S1. (a) The synthetic route of SPMA. (b) ^1H NMR spectrum of SPMA monomer (solvent $CDCl_3$, 400MHz).

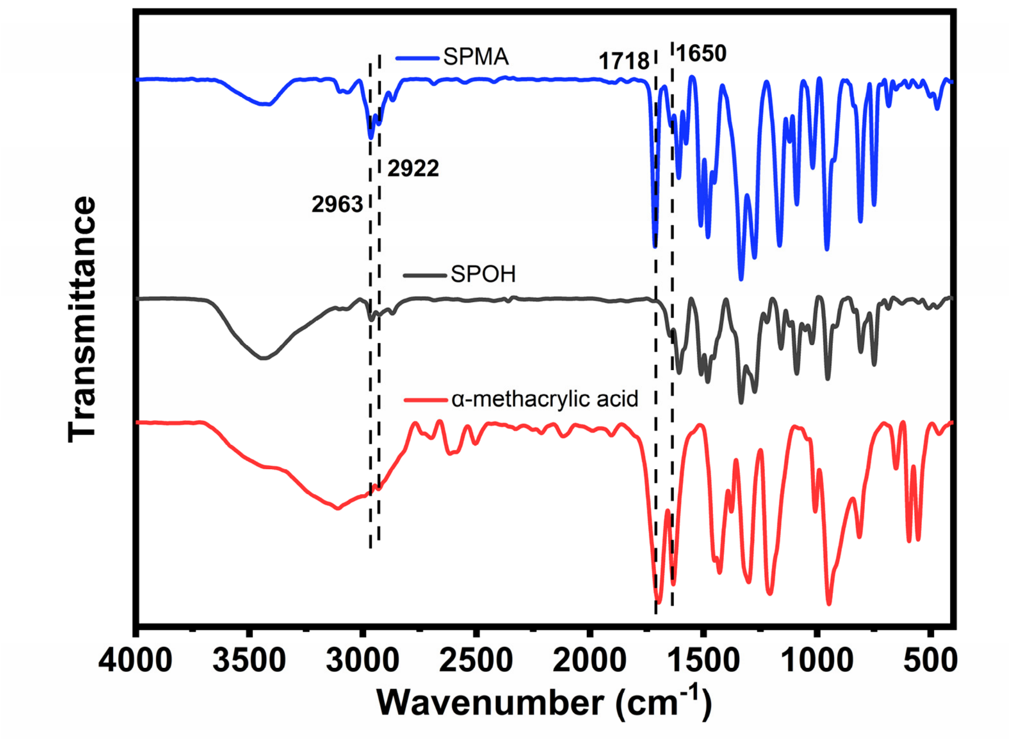

Figure S2. FTIR spectra of SPMA, SPOH and α-methacrylic acid.

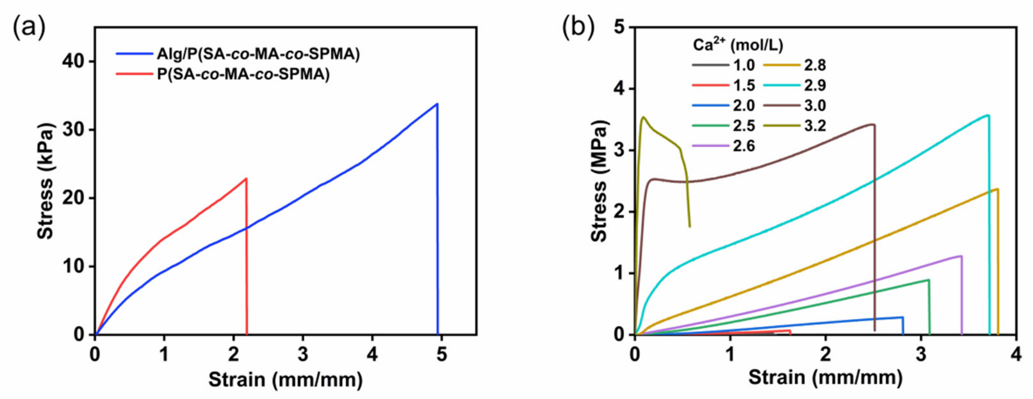

Figure S3. (a) Stress strain curve of P(SA-*co*-MA-*co*-SPMA) hydrogel and Alg/P(SA-*co*-MA-*co*-SPMA) hydrogel. (b) Stress strain curve of Alg/P(SA-*co*-MA-*co*-SPMA)/Ca^{2+} hydrogels soaked in solutions at different Ca^{2+} concentrations.

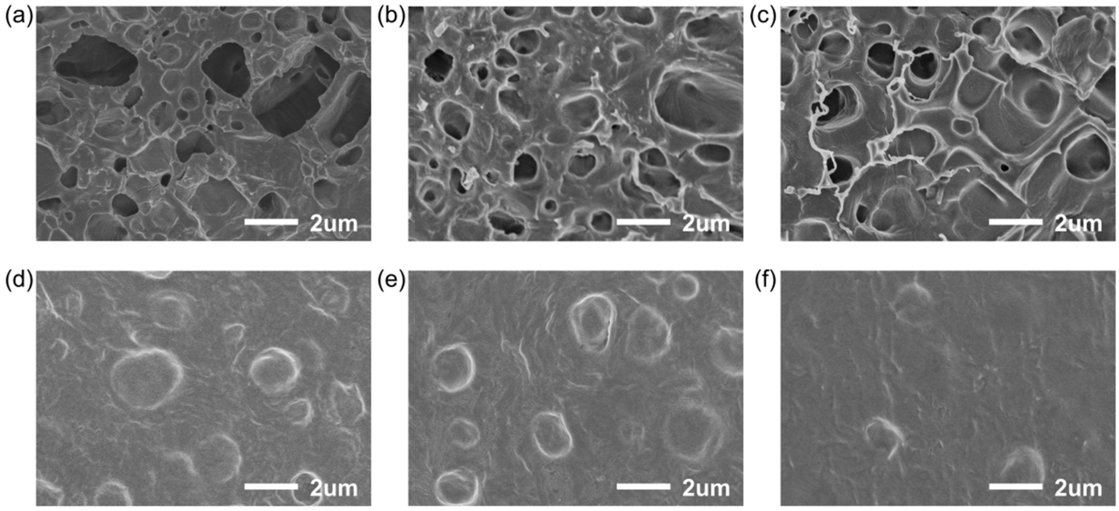

Figure S4. SEM images for cross-sectional morphology of hydrogels (scale bar = 2 μm). (a) P(SA-*co*-MA-*co*-SPMA) hydrogel. (b) Alg/P(SA-*co*-MA-*co*-SPMA) hydrogel. (c) Alg/P(SA-*co*-MA-*co*-SPMA)/$Ca^{2+}_{2.0M}$ hydrogel. (d) Alg/P(SA-*co*-MA-*co*-SPMA)/$Ca^{2+}_{2.6M}$ hydrogel. (e) Alg/P(SA-*co*-MA-*co*-SPMA)/$Ca^{2+}_{2.8M}$ hydrogel. (f) Alg/P(SA-*co*-MA-*co*-SPMA)/$Ca^{2+}_{3.0M}$ hydrogel.

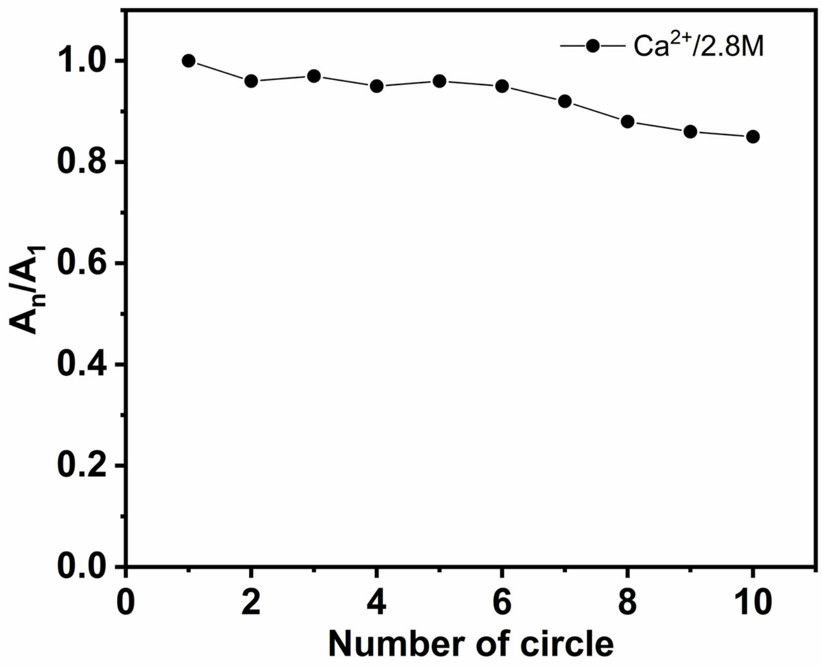

Figure S5. Changes of A_n/A_1 during repeated coloration/discolorization cycle of Alg/P(SA-*co*-MA-*co*-SPMA)/$Ca^{2+}_{2.8M}$ hydrogel irradiated by alternative UV/vis light, where A_1 and A_n represent the intensities for the first and nth cycles at the maximum absorption wavelength.

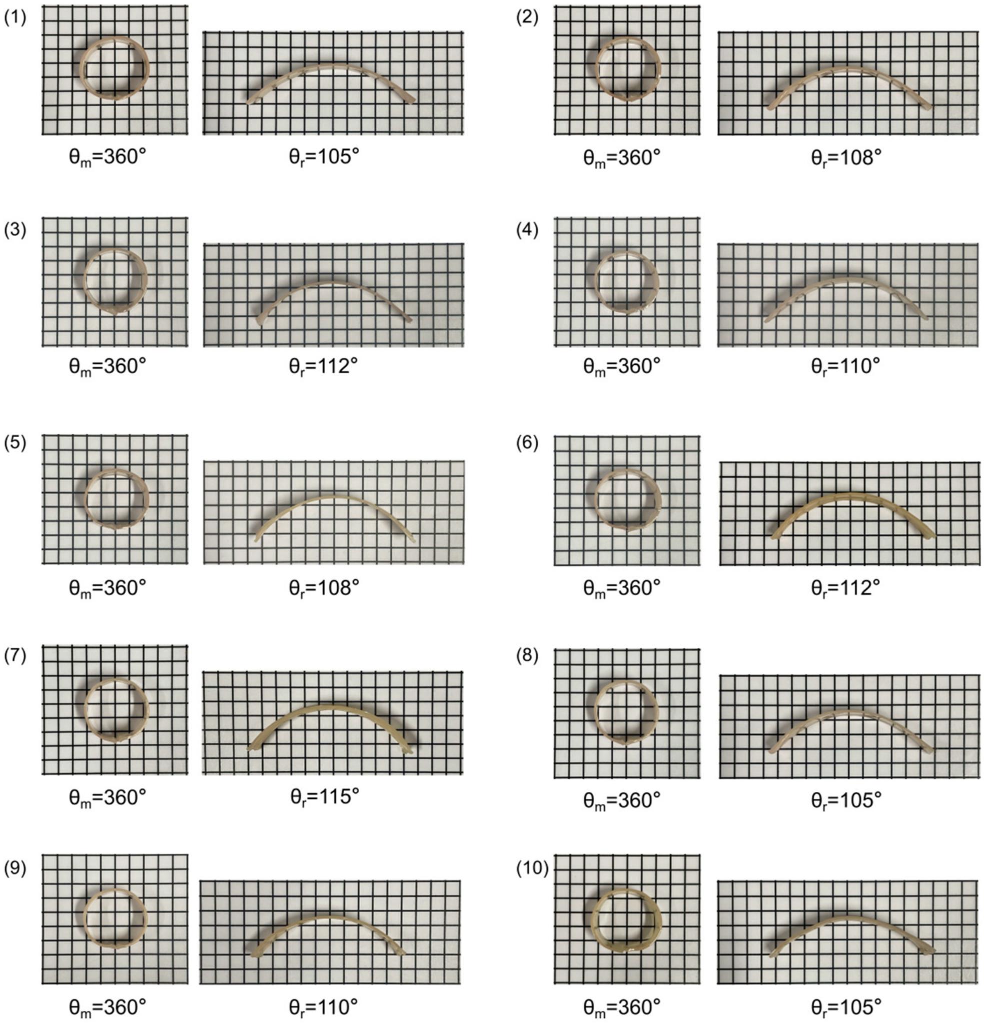

Figure S6. The photos of Alg/P(SA-*co*-MA-*co*-SPMA)/$Ca^{2+}_{2.8M}$ hydrogel conducting 10 cycles of shape fixation and shape recovery.

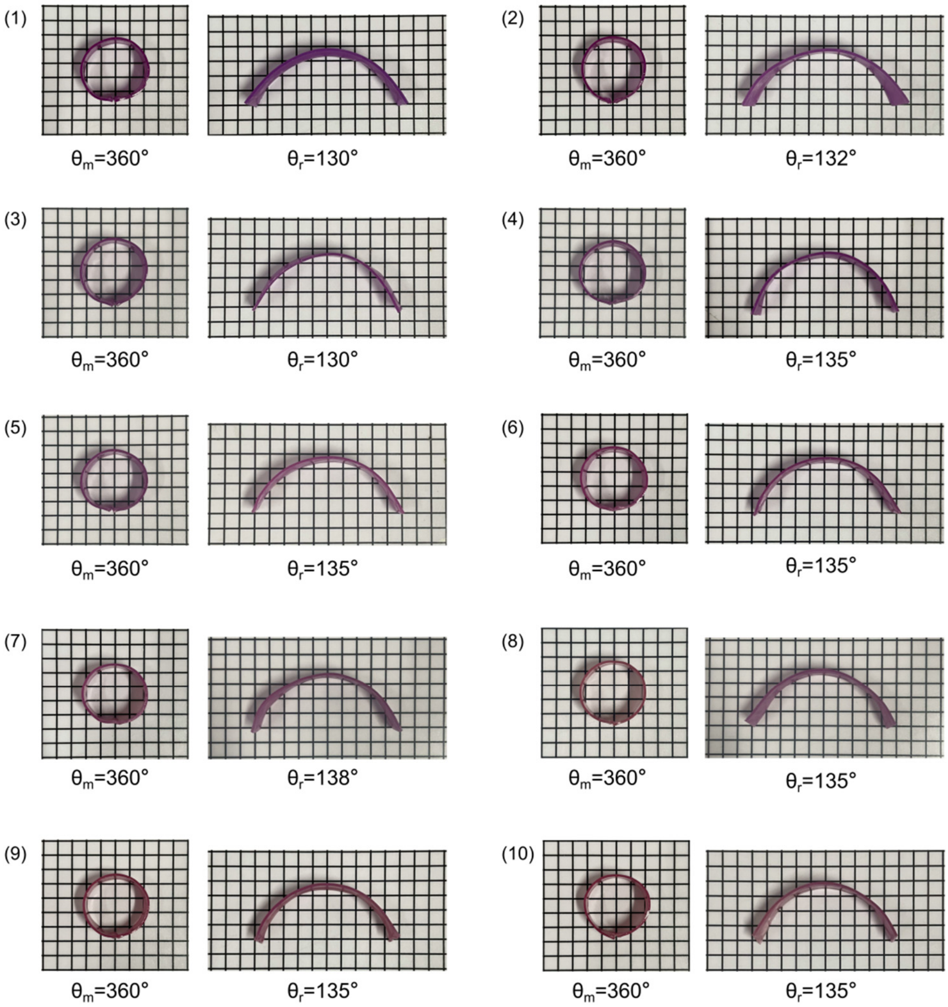

Figure S7. The photos of Alg/P(SA-*co*-MA-*co*-SPMA)/$Ca^{2+}_{2.8M}$ hydrogel conducting 10 cycles of shape fixation and shape recovery under UV irradiation.
